# A Versatile, Bar-Coded Nuclear Marker/Reporter for Live Cell Fluorescent and Multiplexed High Content Imaging

**DOI:** 10.1371/journal.pone.0063286

**Published:** 2013-05-14

**Authors:** Irina Krylova, Rachit R. Kumar, Eric M. Kofoed, Fred Schaufele

**Affiliations:** Center for Reproductive Sciences, University of California San Francisco, San Francisco, California, United States of America; Broad Institute of Harvard and MIT, United States of America

## Abstract

The screening of large numbers of compounds or siRNAs is a mainstay of both academic and pharmaceutical research. Most screens test those interventions against a single biochemical or cellular output whereas recording multiple complementary outputs may be more biologically relevant. High throughput, multi-channel fluorescence microscopy permits multiple outputs to be quantified in specific cellular subcompartments. However, the number of distinct fluorescent outputs available remains limited. Here, we describe a cellular bar-code technology in which multiple cell-based assays are combined in one well after which each assay is distinguished by fluorescence microscopy. The technology uses the unique fluorescent properties of assay-specific markers comprised of distinct combinations of different ‘red’ fluorescent proteins sandwiched around a nuclear localization signal. The bar-code markers are excited by a common wavelength of light but distinguished ratiometrically by their differing relative fluorescence in two emission channels. Targeting the bar-code to cell nuclei enables individual cells expressing distinguishable markers to be readily separated by standard image analysis programs. We validated the method by showing that the unique responses of different cell-based assays to specific drugs are retained when three assays are co-plated and separated by the bar-code. Based upon those studies, we discuss a roadmap in which even more assays may be combined in a well. The ability to analyze multiple assays simultaneously will enable screens that better identify, characterize and distinguish hits according to multiple biologically or clinically relevant criteria. These capabilities also enable the re-creation of complex mixtures of cell types that is emerging as a central area of interest in many fields.

## Introduction

The maturation of screening capabilities over the past two decades has been realized through the progressive miniaturization of assays that has led to an increase in the number of compounds that can be screened [1]. Today, a major impediment to improved screening centers on the design of assays with appropriate biologic or clinical relevance [1]–[3]. One way to improve the biological significance of a screening project is to screen several biologically relevant or related assays in parallel. However, conducting screens against multiple independent assays multiplies the time and cost of screening. These considerations have led to an emphasis on maximizing the information collected within one primary screening assay.

For cell-based screens, high throughput fluorescence microscopy is sometimes used to increase content within the primary assay [4]. Multiple components are stained with unique fluorophores allowing the amounts of each factor to be quantified in relationship to their cellular and/or subcellular distributions [5]–[8]. This ‘high content analysis’ (HCA) approach can improve the quality of the screen provided that the added parameters measured are biologically relevant. However, overlap in the excitation and emission properties of fluorophores limits the number of distinct fluorescent channels available for fluorescence imaging [9] and each additional channel slows collection speed. Furthermore, one or two of those fluorescent channels typically are used for marking specific cellular structures necessary to enable the automated image segmentation required to analyze the data [5], [10]–[11]. Overall, improved technologies that allow multiple assays to be combined in a single well and distinguished following rapid collection would improve screening efficiency and relevance [12].

*In vitro*, different biochemical assays may be combined and incubated together with a drug if each assay is loaded onto beads of unique shapes or sizes that can readily be distinguished [13]–[14]. However, the biologic and/or clinical relevance of a screen often relies on performing the assays within cellular environments pertinent to function [7], [15]. Therefore, the ability to apply advanced multiplexing capabilities for cell-based assays would be advantageous to many screening studies. Some screens also would benefit from the ability to re-sample, over time in live cells, fluorescent protein (FP)-based reporters of function [16]. In some cases, live cell assays also can improve hit identification by minimizing sample processing and staining which sometimes introduces well-to-well variability.

Our objective was to create a live-cell screening paradigm in which multiple cell-based assays could be combined in a single well and distinguished by automated microscopy using a limited number of fluorescent channels. The bar-coded markers developed were designed to match the needs of high throughput image segmentation and quantitative analyses. The bar-code consists of a series of nuclear, red fluorescent markers that can all be excited with a common excitation wavelength but distinguished ratiometrically by two emission channels. This enables distinctly marked cell lines to be distinguished without a loss in screening speed. We also combined the bar-code with different yellow fluorescent protein (YFP)-based reporters to demonstrate the effectiveness of the bar-code in distinguishing co-cultured YFP-based assays with different responses to an added drug. We suggest ways in which multiple bar-coded, cell-based assays may be distinguished by microscopy within a single well using only two fluorescence channels.

## Results

### A Nuclear FP_NLS_FP Marker for Live-Cell Microscopy

High throughput microscopy depends on the automated identification of cellular structures in all images by analysis algorithms that group together collections of pixels showing intensity, size or shape characteristics typical of that structure [10]–[11], [17]–[18]. Because the nuclei of cells growing as a monolayer in a multi-well dish usually are well-separated, ‘segmentation’ protocols that define individual nuclei tend to be more successful than protocols that define other subcellular structures. Once individual nuclei are defined using a nuclear marker, the cell margins and/or other substructures associated with that nucleus can be identified by searching for other fluorescent markers surrounding the marked nucleus.

For fluorescence microscopy studies, the nuclei of fixed cells can be identified following staining with any of a number of fluorescent dyes [19]. Some fluorescent dyes are able to stain live cells. However, those dyes show significant cytotoxicity that prevents their application to live cell studies longer than one-to-two hours. Long-term imaging of live cells must overcome this toxicity [20]. The labeling of live cell nuclei can be accomplished by fusing fluorescent proteins (FPs) to a nuclear protein, such as a histone or a lamin [21]–[22], but adding a FP to centrally important nuclear proteins could affect normal cell function in unknown ways. Nuclear localization sequences (NLS) have been fused to a FP [23] which can create less disruptive nuclear markers. Adding only a single, few amino acid-long, NLS to an FP is not sufficient for nuclear retention, presumably because the small size of the FP_NLS_ fusion allows it to freely move in and out of the nucleus [24]. Adding multiple NLSs to an FP improves nuclear translocation [25] but those markers often target to specific locations within the nucleus [23], [26] creating bright fluorescent areas can impair optimal segmentation of nuclei.

**Figure 1:**
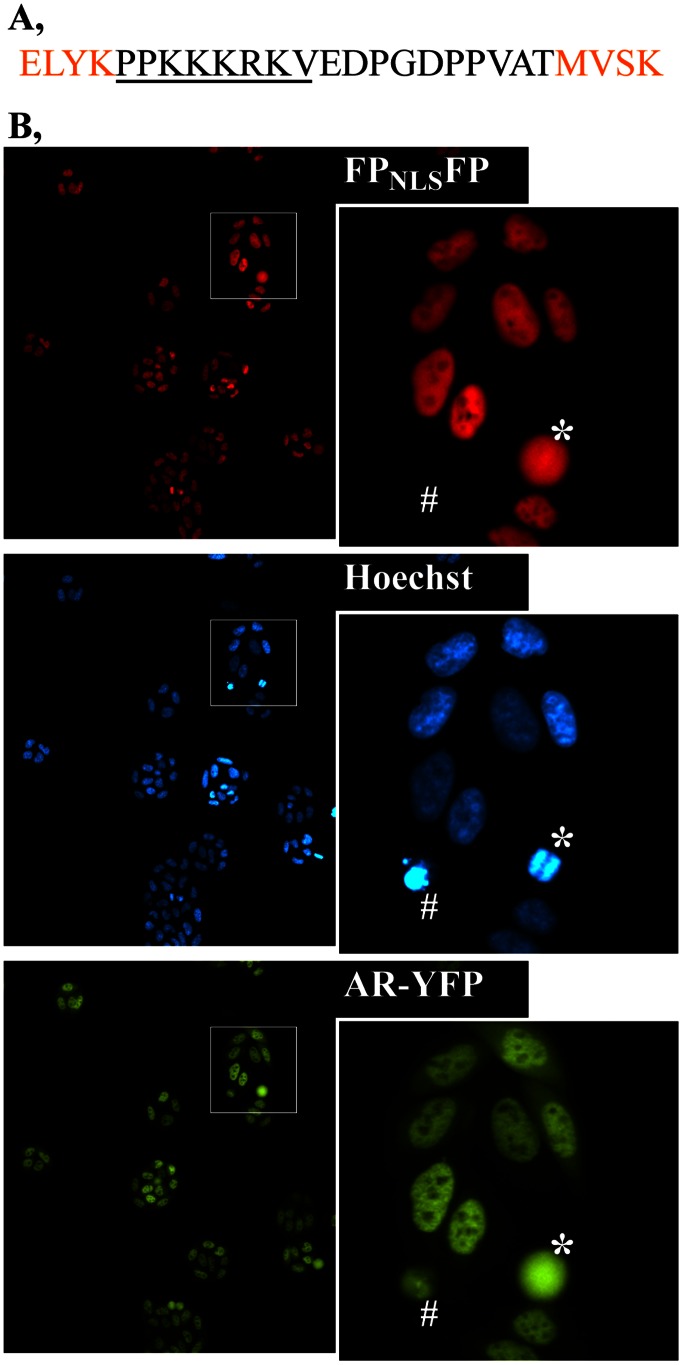
Construction and utility of the FP_NLS_FP nuclear marker. **A,** Amino acids inserted (black font, SV40 NLS underlined) between two mCherry FPs (orange font) within the mCherry_NLS_mCherry nuclear marker. The insertion sequence and location is similar for the other FP_NLS_FP nuclear markers created in this study. **B,** Nuclear fluorescence of the mCherry_NLS_mCherry marker stably expressed in a HeLa cell line in relationship to nuclei stained with Hoechst 33342. The locations of a YFP-tagged Androgen Receptor (AR) co-expressed in this cell line also are shown. The cells were grown in media containing testosterone, which translocates the AR into the cell nuclei. Images were captured with a 10x objective. *, mitotic cell. #, dying cell.

We created a live-cell ‘FP_NLS_FP’ nuclear marker by sandwiching two FPs around the NLS of the simian virus 40 (SV40) T antigen. The sequence of the junction between the FPs is shown in Fig. 1A, with the NLS underlined. A mCherry_NLS_mCherry fusion protein marked the cell nuclei upon expression in HeLa cells (Fig. 1B) or other cells (see later figures). By comparison to the more mottled appearance of nuclei stained with the DNA-binding dye Hoechst 33342, the FP_NLS_FP nuclear marker evenly distributed throughout the nucleoplasm although it was less abundant in nucleoli.

The FP_NLS_FP protein differed from DNA-binding chemicals in how it marked certain types of nuclei. Whereas Hoechst 33342 marked two sets of condensed chromatin in mitotic nuclei, FP_NLS_FP remained distributed in the nucleus (Fig. 1B, *). Dying cells that retained DNA were stained with Hoechst 33342 but showed no FP_NLS_FP fluorescence (Fig. 1B, #) presumably because the nuclear envelope confining the FP_NLS_FP was not intact. In the same cell, a YFP-linked DNA-binding transcription factor also marked the remnant DNA (Fig. 1B, AR-YFP #). The FP_NLS_FP marker therefore seemed to behave as a non-DNA-binding factor imported into intact nuclei where it distributed throughout much of the nucleoplasm. Below we characterize its utility as a live cell segmentation marker after which we describe variations on the marker for use as a bar-code in multiplexed high throughput analyses.

### The FP_NLS_FP Marker is Competent for High Throughput Segmentation

**Figure 2:**
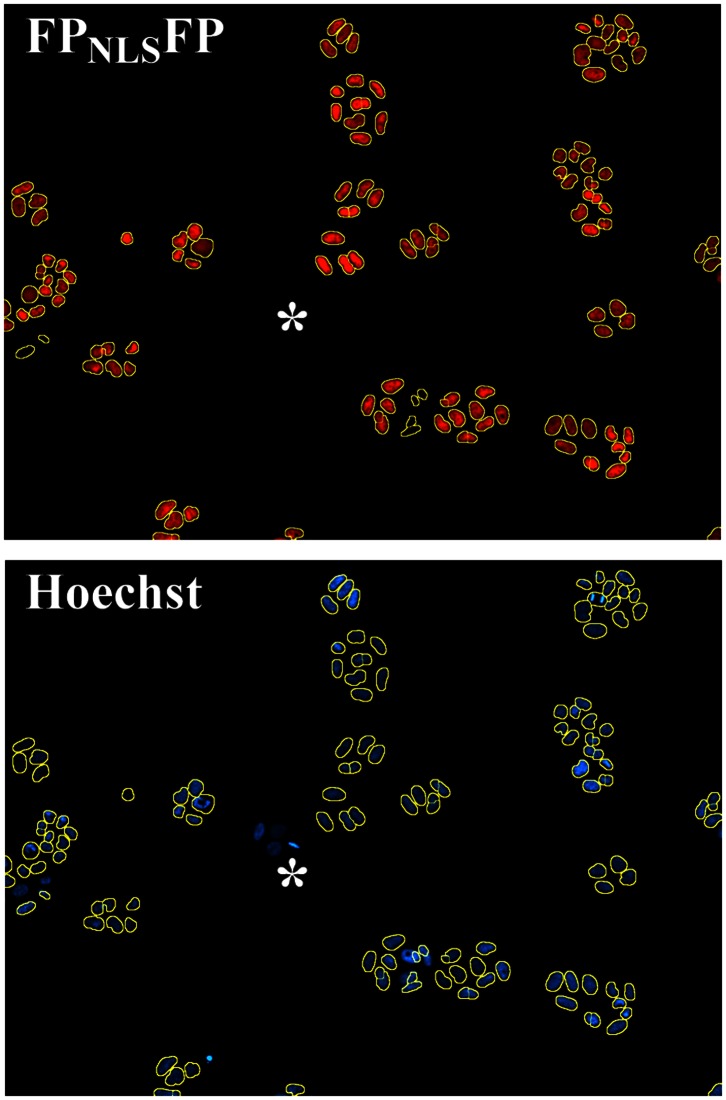
Segmentation of cell nuclei marked with FP_NLS_FP. The boundaries of objects with contiguous FP_NLS_FP expression established by a commercial analysis software (yellow circles) marked the boundaries of nuclei stained with the DNA binding dye Hoechst 33342. *, colony of cells in which FP_NLS_FP expression is lost sporadically.

A commercially available segmentation program defined boundaries for FP_NLS_FP- marked objects (Fig. 2, yellow lines) that were similar to the margins of Hoechst-stained nuclei. Quantitatively, 99.6% of 426,326 mCherry_NLS_mCherry-marked objects identified from a stable HeLa cell line were scored by automated image segmentation as counterstained with Hoechst 33342. Of eight ‘red’ FP_NLS_FP-expressing cell lines assessed to date, all showed Hoechst 33342 counterstaining of >98.5% of FP_NLS_FP-marked objects. Thus, the FP_NLS_FP protein accurately marked nuclei. Visual inspection showed that the FP_NLS_FP-marked objects that did not counterstain with Hoechst 33342 tended to be pieces of low intensity, red-fluorescent debris.

In all stable cell lines examined, some Hoechst-stained nuclei did not show any FP_NLS_FP expression. Since there is no selective advantage to continued FP_NLS_FP expression in stable cell lines, cells that lose FP_NLS_FP expression divide into Hoechst-stained FP_NLS_FP negative ‘colonies’ (Fig. 2, *). For the stable cell lines described here, the selectable marker and the FP_NLS_FP expression cassette were co-introduced on separate plasmids. Introducing the FP_NLS_FP and antibiotic resistance expression cassettes combined within a single vector may improve the maintenance of FP_NLS_FP expression under antibiotic selection pressure.

Green FPs have been shown to be somewhat toxic to cells when expressed and activated by its excitation light [27]–[28]. If the red FP_NLS_FP created for the current study were toxic to the cells, that toxicity could contribute to the overgrowth of the culture by faster growing cells that sporadically lose FP_NLS_FP expression. To examine if FP_NLS_FP expression inhibited cell viability and growth, FP_NLS_FP-marked prostate cancer cell lines were cultured for >25 passages to obtain mixed populations of FP_NLS_FP-positive and -negative cells in the same culture. Table 1 shows one representative study comparing the growth of FP_NLS_FP-positive and -negative cells within each cell line; we use this ‘within-subclone’ comparison rather than comparing to a parental cell line since each subclone tends to exhibit slightly different growth characteristics.

**Table 1:** Growth properties of a FP_NLS_FP-tagged LNCaP-C4-2 prostate cancer cell line.

	Total CellNumber	FP_NLS_FP-Positive cells			FP_NLS_FP-Negative cells		
		Cell Number	Fluoresc Intensity	Day 4/0	Cell Number	Fluoresc Intensity	Day 4/0
Day 0	673+/−98	359+/−57	63+/−7	−	314+/−50	2+/−1	−
Day 4	1229+/−140	638+/−75	76+/−9	1.78	591+/−95	1+/−1	1.88
	1218+/−139	632+/−88	77+/−8	1.76	586+/−74	1+/−1	1.87
*	1207+/−152	608+/−77	72+/−8	1.69	599+/−92	0+/−1	1.91
*	1281+/−200	655+/−104	74+/−8	1.82	626+/−111	0+/−0	2.00

Hoechst-stained nuclei are counted on Day 0 and, on replicate plates, 4 Days later.

* These plates were exposed to FP_NLS_FP excitation light on Day 0 to establish if light exposure altered growth of FP_NLS_FP-positive or FP_NLS_FP-negative cells.

Cells were seeded into 384-well dishes and let attach for 2–3 days. The average baseline number of Hoechst 33342-stained nuclei (Table 1, Day 0) was determined by automated microscopy at low (4x) magnification, which covers most of the well. FP_NLS_FP fluorescence (excitation with 560–590 nm, emissions collected at 635–675 nm) also was collected concurrently to establish which cells were FP_NLS_FP-positive or FP_NLS_FP-negative at Day 0. Replicate plates (not yet stained with Hoechst) were maintained in the incubator for an additional four days. On Day 4, FP_NLS_FP-positive and FP_NLS_FP-negative cells were counted in the replicate plates under Hoechst-staining and image collection conditions identical to those used on Day 0. The numbers of cells counted on Day 4 were compared to the numbers counted on Day 0 (Table 1, Day 4/0) to establish the growth rates of the FP_NLS_FP-positive and FP_NLS_FP-negative populations. Some plates were exposed for 500 ms of FP_NLS_FP excitation light on Day 0 (Table 1, *); neither FP_NLS_FP-positive nor -negative cell numbers were affected by that exposure further showing that FP_NLS_FP expression was not grossly toxic to the cells.

For three different prostate cancer cell subclones examined in a total of nine separate studies, the growth rate of the FP_NLS_FP-positive subpopulation averaged 102+/−51% that of the FP_NLS_FP-negative cells. The considerable variation noted amongst the studies likely reflects the sporadic nature of the genetic deletions removing FP_NLS_FP expression and sometimes other factors regulating cell growth. On average though, expression of the FP_NLS_FP marker did not confer any consistent change in the growth to these cell lines, even if photoactivated early in the growth stage.

### Longitudinal FP_NLS_FP Imaging Improves Proliferation Measurement

We next examined how well the FP_NLS_FP marker compared with Hoechst 33342 staining under experimental conditions. Human tumor-derived cell lines remain the most widely used preclinical models to screen for drug candidates exhibiting specific anti-tumor activity [15]. A number of cell-based assays are available to measure cell growth and compound cytotoxicity. Many of these analyses require cell lysis, which can introduce inconsistencies that degrade well-to-well reproducibility. Some of those commonly used high throughput endpoint assays assess metabolic state of the cells rather than cell number and/or viability [29] and therefore may provide hits irrelevant to growth. Direct counting of cell numbers within each well would be preferred for detecting growth (this section) and death (next section).

The following studies were focused on prostate cancer, particularly the most clinically vexing aspect of the disease in which a ‘castration-resistant’ tumor continues to grow even though the patient receives treatments to lower the tumor-promoting actions of androgens (testosterone) acting through the androgen receptor (AR). The growth of LNCaP-C4-2 human prostate cancer cells was investigated because these cells grow slowly in the absence of androgens although growth still is accelerated when androgens are provided [30]. This cell line therefore models both castration-resistant and androgen-regulated prostate cancer cell growth. Growth measurements that improve the reliable identification of wells treated with agents that block the slow, difficult to measure castration-resistant growth of LNCaP-C4-2 may help to define new treatments for that disease.

**Figure 3:**
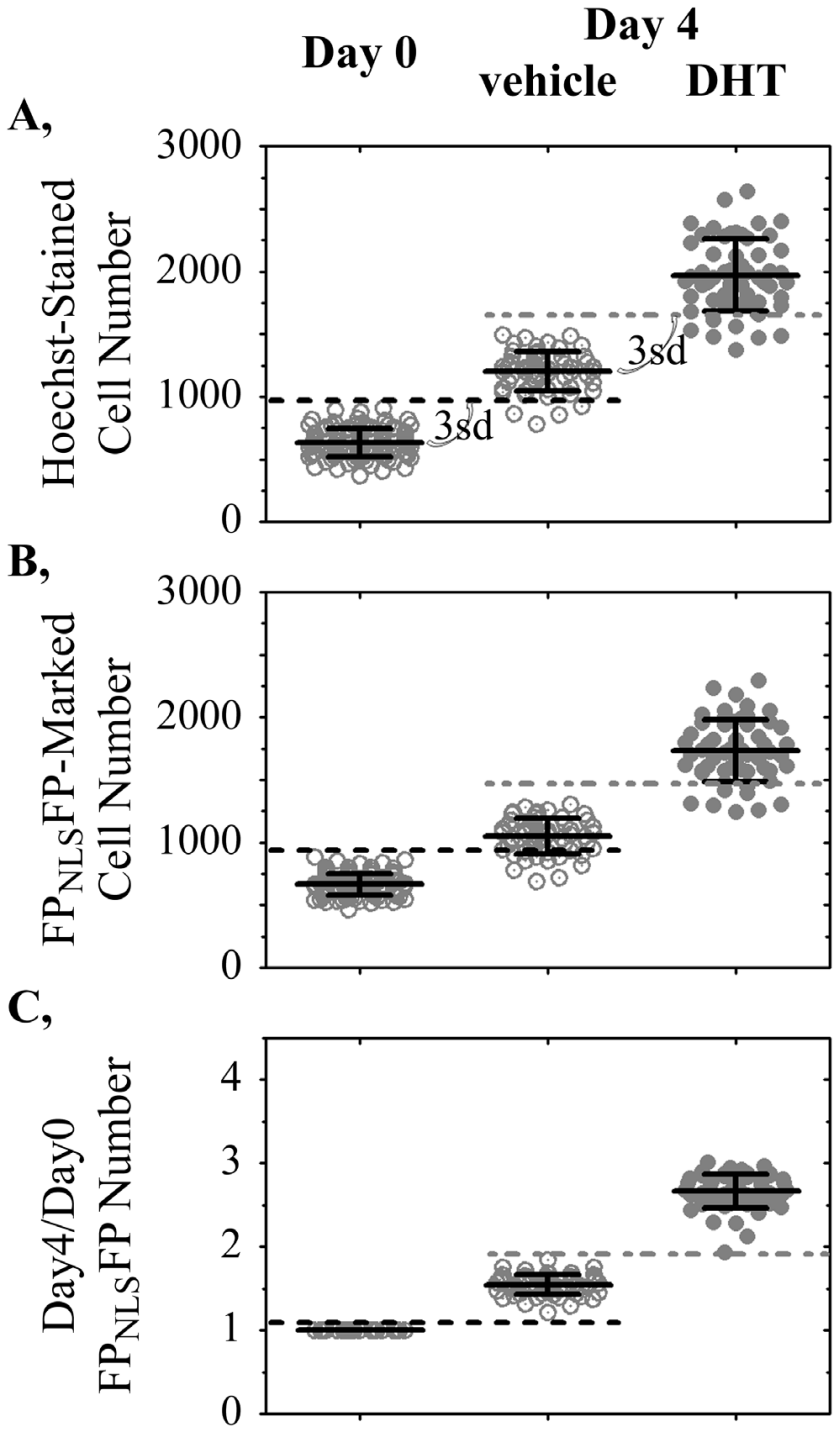
Improved well-to-well reproducibility in cell growth measurement enabled by the FP_NLS_FP live cell nuclear marker. Variations in cell numbers plated in each well (Day 0) obscured the ability to reliably detect an increase in cell number after four days of slow growth by LNCaP-C4-2 prostate cancer cells treated with vehicle or 0.2 nM DHT. Each symbol represents the numbers of **A,** Hoechst 33342-stained nuclei or **B,** FP_NLS_FP-marked nuclei segmented in each well. **C,** Dividing the number of FP_NLS_FP-marked cells on Day 4 by the baseline (Day 0) number of FP_NLS_FP-marked cells in the same well improved the reproducibility of growth measurement. Dotted lines, three standard deviations (3sd) above the mean Day 0 (black dotted line) or Day 4 vehicle-treated (gray dotted line) measurements are shown. The 3sd cut-offs were used to determine the number of, respectively, vehicle-treated and DHT-treated wells that were scored falsely in the Day 0 and vehicle-treated wells.

For high throughput screening, an assay that shows minimal well-to-well variation in output is essential. Otherwise, a drug that changes the assay in a single well (screening typically is done without replicates) can easily be lost within the well-to-well measurement noise. Figure 3A shows the Day 0 counts of Hoechst 33342-stained cells in multiple control wells in comparison to Day 4 cell counts of Hoechst-stained cells in other wells treated with vehicle or 0.2 nM dihydrotestosterone (DHT). The slow growth of LNCaP-C4-2 cells in the absence of testosterone (Fig. 3A, vehicle) was difficult to measure reliably in every well because growth was slow in relationship to variations in the counting of cell numbers (Fig. 3A, Day 0). The dotted black line (Fig. 3A) depicts three standard deviations above the average cell counts on Day 0. Many wells on Day 4 had cell counts below that threshold and therefore would be indistinguishable from wells in which there was little to no growth. Screens for agents that block the slow castration-resistant growth of LNCaP-C4-2 cells therefore could identify an unacceptably large number of false negative wells in which apparent growth-inhibition is only a measurement anomaly. Parallel wells treated with DHT grew faster but also were difficult to reliably distinguish from those treated with vehicle alone (Fig. 3A, dotted gray line).

If the cell counts on Day 4 could be normalized for the variations in the numbers of cells present within each well on Day 0, it might be possible to improve the well-to-well reproducibility of growth measurement. Because FP_NLS_FP live cell markers can be imaged repetitively, the numbers of FP_NLS_FP-marked cells counted in one well on Day 0 can be compared directly to the cell numbers counted in that same well at a later time-point. We therefore created LNCaP-C4-2 cell lines that stably expressed a FP_NLS_FP nuclear marker. Figure 3B shows the numbers of FP_NLS_FP-marked cells measured in all wells at Day 0. The same wells were treated with vehicle or 0.2 nM DHT and imaged four days later. As with cell counting by Hoechst 33342-staining, if considering only the Day 4 and Day 0 average numbers of cells in each well, the variation in the numbers of cells plated obscured the ability to reliably score cell growth (Fig. 3B, Day 4). However, the FP_NLS_FP live cell nuclear marker enabled the number of cells within each well counted on Day 4 to be compared to the Day 0 baseline cell number within the same well (Fig. 3C, Day4/Day0). That longitudinal measurement improved noticeably the ability to reliably detect, in all wells, the slow castration-resistant growth of the FP_NLS_FP-tagged LNCaP-C4-2 cell line.

The improved accuracy of longitudinal cell counting is shown in Table 2 for three FP_NLS_FP-marked cell lines. When the numbers of cells stained by Hoechst 33342 at Day 4 were compared to the average number of cells in control wells stained similarly on Day 0, anywhere from 7 to 34% of DHT-treated wells were scored as not being activated by DHT (Table 2, ‘% False Negative…Hoechst’ column). One cell line (that shown in Fig. 3) also had 7% of wells in the absence of testosterone scored as not growing. By contrast, when the numbers of FP_NLS_FP-marked cells counted on Day 4 were normalized to the Day 0 cell counts from the exact same well (Table 2, right column), the number of false negatives was minimized for all cell lines. The FP_NLS_FP-marked nuclei counted on Day 0 thus internally controlled for well-to-well variations in the numbers of cells plated into each well to improve the reliability of cell growth measurements within any single well.

**Table 2:** Longitudinal comparison of FP_NLS_FP-marked cell numbers in the same well on Day 4 and Day 0 minimizes incorrect growth measurements of FP_NLS_FP-tagged LNCaP-C4-2 cell lines.

CellSubclone	Treatment	Fold Growthover Day 0	FP_NLS_FP fluorescentintensity	% False Negative Cells Counted byHoechst 33342 Staining	% False Negative Cells Countedby Longitudinal FP_NLS_FP Marking
E01	vehicle	2.21	63+/−7	0%	0%
	DHT	2.91		7%	0%
H	vehicle	1.55	283+/−18	7%	0%
	DHT	2.67		14%	0%
F12	vehicle	2.53	68+/−5	0%	0%
	DHT	3.64		34%	0%

‘vehicle’ false negatives indicates wells scored as having no growth from Day 0 to Day 4 (fewer cells than 3 standard deviations above the Day 0 cell numbers).

‘DHT’ false negatives indicates wells scored as having no DHT-activated growth (fewer cells than 3 standard deviations above the Day 4 vehicle cell numbers.

### Ratiometric Bar-Coding to Expand Fluorescent Imaging

**Figure 4:**
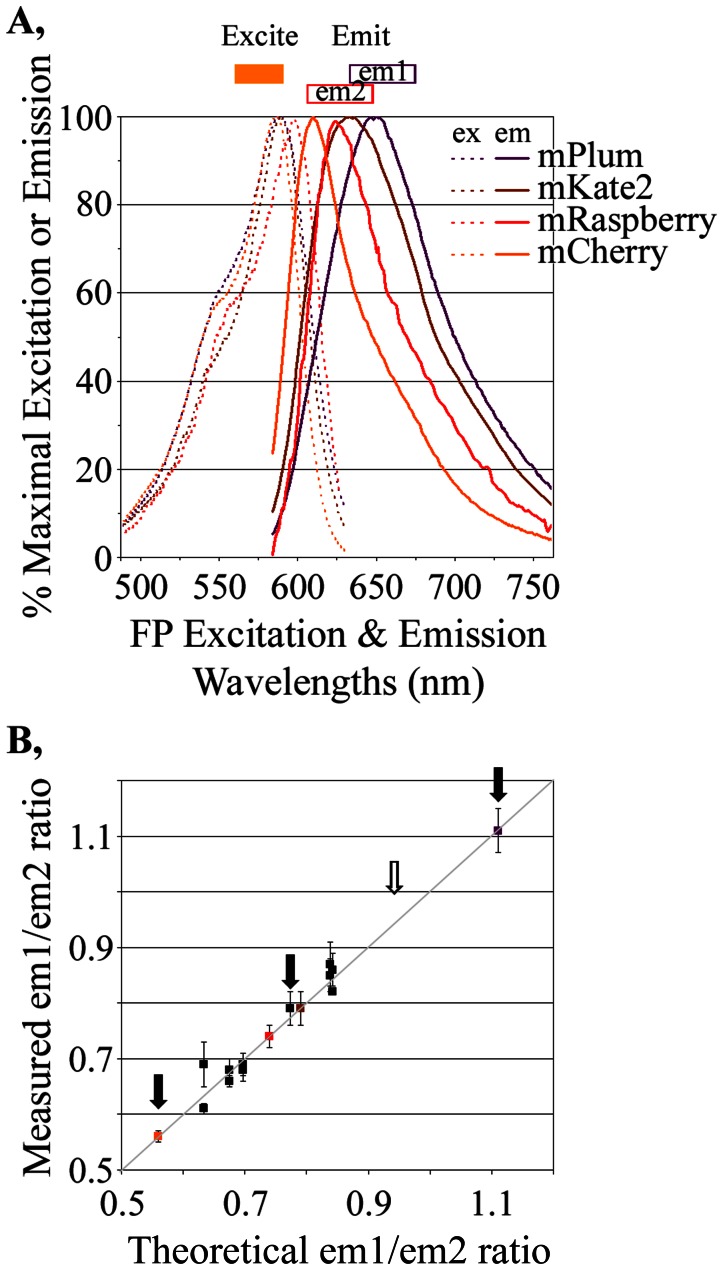
Cellular “bar-coding”. **A,** Excitation and emission properties of four FPs used to create the bar-code. All FPs were excited by light of 560–590 nm (orange box) but emitted different relative amounts in two emission channels (em1: 635–675 nm; em2: 608–648 nm). For example, the area under the curve collected for mPlum in em1 would be slightly more than that in em2 whereas, for mCherry, em2 emissions would be much higher than em1 emissions. These differences were seen in practice (Table 3). **B,** The measured em1/em2 ratios for sixteen FP_NLS_FP markers using all possible combinations of the four FPs (Table 4) were similar to those predicted if one assumes no FRET amongst the FPs. The theoretical em1/em2 ratios were calculated from their relative abilities to be excited by 560–590 nm light (Fig. 4A), their relative brightness once excited (Table 3) and the em1 and em2 emissions detected by our instrument for the four homogeneous FP_NLS_FPs (mPlum_NLS_mPlum, mCherry_NLS_mCherry etc).

The cell lines shown in Table 2 were marked with slightly different variations of the FP_NLS_FP marker (‘E01’ with mRaspberry_NLS_mKate2, ‘H” with mCherry_NLS_mCherry and ‘F12’ with mPlum_NLS_mPlum). However, their overlapping excitation and emission spectra (Fig. 4A) permitted all to be excited with 560–590 nm light (Fig. 4, orange bar) and detected by the collection of 635–675 nm emissions (Fig. 4A, em1). This ability to image a series of different red fluorescent markers with overlapping, but distinct, spectral characteristics formed the foundation for a ‘bar-code’ under which distinct fluorophores could be distinguished by their unique emission properties. Once excited by 560–590 nm light, the fluorescence emitted from the distinct FPs is collected in two different emission channels (Fig. 4A, em1: 635–675 nm; em2: 608–648 nm). Some red FPs will emit more light in em1 than em2 and others will emit more in em2 than em1. Thus, each FP has a characteristic ratio in the amounts of background-subtracted fluorescence in the em1 channel relative to the em2 channel. The key to applying these ratios for distinguishing the different FPs is to establish how accurately those ratios can be measured.

To test how well this theory works in practice, four red FPs (mPlum, mKate2, mRaspberry and mCherry) were transiently expressed in CHO cells and their relative, background-subtracted fluorescence levels in the em1 and em2 channels were determined (Table 3, measured em1/em2). We chose those FPs because of their spectral (Fig. 4A) and physical properties (Table 3). Each FP showed characteristic, reproducibly measured em1/em2 ratios. The measured em1/em2 ratios closely approximated the theoretical em1/em2 ratios (Table 3) expected by calculating the area under the emission curves (Fig. 4A) in the em1 (635–675 nm) and em2 (608–648 nm) channels, corrected for slight differences (obtained from technical data sheets) in the absorption of emitted light by the em1 and em2 filters and in the different abilities of our microscope optics/camera to absorb/detect emissions in em1 and em2 (estimated empirically by instrument calibration at 89.3% in em1 compared to em2). Thus, the distinct emission characteristics of the different FPs coincided in living cells with their known properties measured *in vitro*.

**Table 3:** em1 (635–675 nm) fluorescence channel relative to em2 (608–648 nm) of the indicated red FPs.

	mPlum	mKate2	mRaspberry	mCherry
measured em1/em2, mean +/− sd	1.07+/−0.07	0.80+/−0.05	0.66+/−0.03	0.55+/−0.01
(number of cells measured)	(n = 414)	(n = 349)	(n = 175)	(n = 126)
theoretical em1/em2 ratio	1.06	0.82	0.67	0.54
Physical Properties [57]				
Ex λmax nm	590	588	598	587
Em λmax nm	649	635	625	615
E_mol_ a	41,000	62,500	86,000	72,000
QY^b^	0.1	0.4	0.15	0.22
Brightness relative to EGFP^c^	13%	79%	41%	50%
t_0.5_ maturation (hours)	1.66	∼0.33	0.92	0.25–0.6

a Molar Extinction Coefficient (M^−1^ cm^−1^): the ability of the fluorophore to absorb light.

b Quantum Yield: proportion of absorbed photons re-emitted as fluorescent photons.

c Product of QY and Emol, relative to that of EGFP (100%).

Ratios determined in 4x images collected on transiently transfected CHO cells.

### A Series of FP_NLS_FP Nuclear Bar-Code Markers

Each FP_NLS_FP nuclear marker has two FPs which can further alter, and potentially expand, the em1/em2 ratios that can be obtained from the four red FP examined. We created a 16-member matrix of all possible combinations of the four fluorophores (Table 4). All 16 FP_NLS_FP markers localized to cell nuclei (not shown) when transiently expressed in CHO cells. The em1/em2 ratios for three of the ‘homogenous’ FP_NLS_FP bar codes (mPlum_NLS_mPlum, mKate2_NLS_mKate2, mCherry_NLS_mCherry, Table 4) agreed well with em1/em2 ratios of their mPlum, mKate2 and mCherry counterparts (Table 3). The em1/em2 ratio for mRaspberry_NLS_mRaspberry (0.74) tended to deviate from its parental mRaspberry FP (0.66) for unknown reasons. The twelve FP_NLS_FP markers that consisted of two different red FPs possessed characteristic em1/em2 fluorescence ratios that, as discussed below, reflected the fluorescence properties of their constituent FPs.

**Table 4:** Bar-Coded FP_NLS_FP Nuclear Markers.

	FP-C:mCherry	FP-C:mRaspberry	FP-C:mKate2	FP-C:mPlum
FP-N: mCherry	0.56+/−0.01	0.61+/−0.01	0.68+/−0.02	0.68+/−0.02
FP-N: mRaspberry	0.69+/−0.04	0.74+/−0.02	0.79+/−0.03	0.82+/−0.00
FP-N: mKate2	0.69+/−0.02	0.79+/−0.03	0.79+/−0.03	0.87+/−0.04
FP-N: mPlum	0.66+/−0.01	0.86+/−0.03	0.85+/−0.03	1.11+/−0.04

Ratio of fluorescence emitted in em1 (635–675 nm) relative to that emitted in em2 (608–648 nm) for the FP-N_NLS_FP-C bar-code vectors. Ratios determined in 10x images collected on transiently transfected CHO cells.

**Figure 5:**
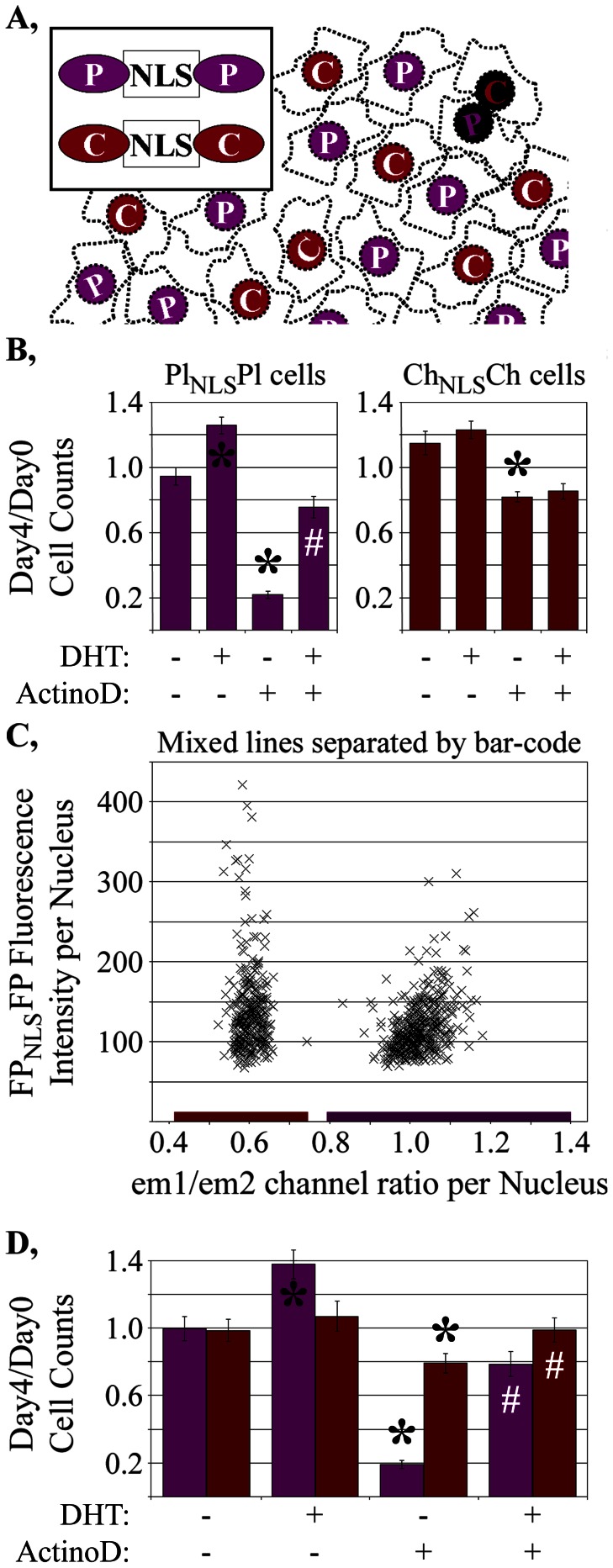
Application of bar-code to cell counting studies. **A,** Concept of bar-code for mixing differentially marked FP_NLS_FP expressing cells. **B,** Differential response of two LNCaP-C4-2 cell subclones to an inhibitor of cell growth (actinomycin D). **C,** em1/em2 ratios of all cells within a representative well (x-axis) compared to the intensities of each cell in the em1 channel. **D,** LNCaP-C4-2 cells mixed, co-plated, treated exactly as in figure 5B then separated according to the bar-code showed similar treatment responses to the individually plated cells. Growth measurements are shown as the mean +/− sd from 8 (Fig. 5B) or 16 (Fig. 5D) wells for each treatment condition. *, statistically significant (p<0.01) increases or decrease in cell number relative to vehicle-treated cells; #, statistically significant (p<0.01) increase in cell number of DHT/actinomycin D treated wells relative to actinomycin D-treated wells.

**Figure 6:**
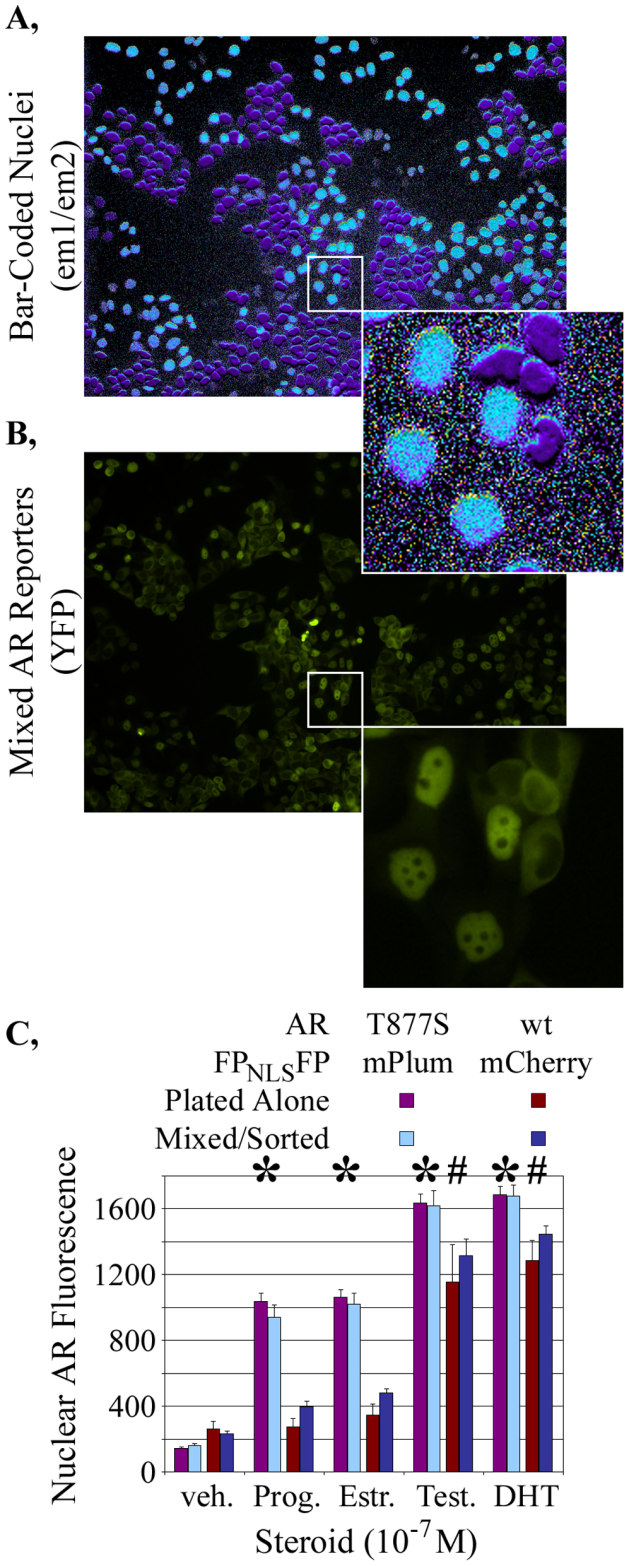
Bar-code separation of different, co-cultured assays. Representative images of **A,** the em1/em2 ratio and **B,** YFP-tagged AR showed the FP_NLS_FP bar code to accurately discriminate between two differentially marked HeLa cell lines in which a wild-type (wt) or mutant (T877S) AR have different nuclear distributions when grown with 10^−8^ M estradiol. **C,** Quantification of nuclear AR levels in the two different cell lines after incubation with 10^−7^ M of the indicated steroids demonstrated that the differential responses between the wt and T877A ARs observed when plated separately were retained when the cell lines were mixed in a well and sorted according to the bar-code. Nuclear AR measurements are shown as the mean +/− sd from 48 wells for each treatment condition. The distinct responses of the two assays to different hormones are indicated by *, # (statistically significant increases, p<0.01, that were at least double the nuclear AR-YFP intensities in vehicle-treated mPlum_NLS_mPlum and mCherry_NLS_mCherry cells, respectively).

**Figure 7:**
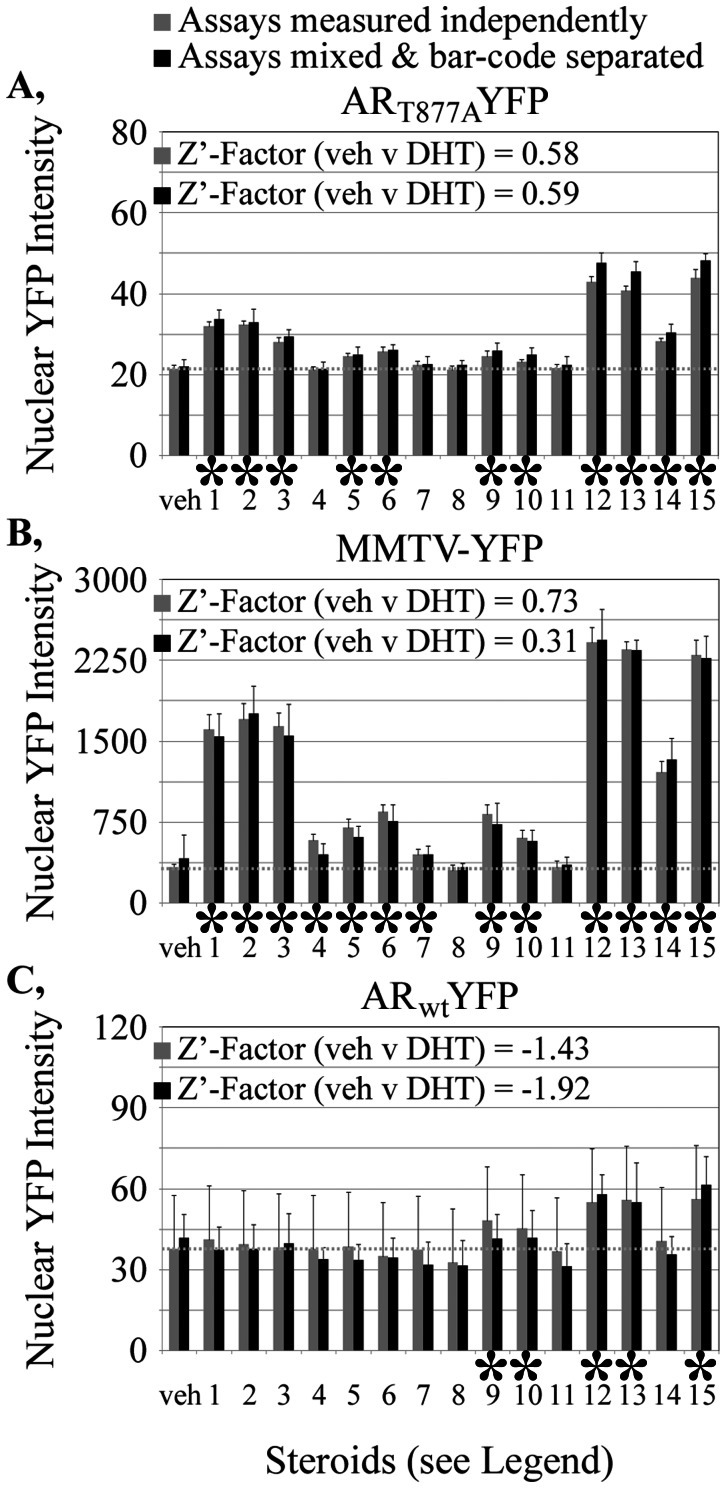
Characterization of three differentially bar-coded LNCaP-C4-2 cell lines, each expressing a different YFP-based reporter assay. **A–C,** YFP fluorescence intensities within the FP_NLS_FP-marked cell nuclei of each separately-cultured reporter line are shown in response to fifteen different steroids (10^−8^ M each). Nuclear YFP measurements are shown as the mean +/− sd from 10 fields for each treatment condition. Gray bars, measurements for indicated assay plated independently into separated wells. Black bars, measurements from wells in which the three assays are co-cultured and separated by the bar-code. The dotted gray line represents the measurements obtained upon treatment of the independent assays with vehicle only. Steroids: 1: pregnenolone, 2: progesterone, 3: 11-deoxycorticosterone, 4: aldosterone, 5: 17-hydroxypregnenolone, 6: 17-hydroxyprogesterone, 7: 11-deoxycortisol, 8: cortisol, 9: dehydroepiandrosterone, 10: androstendione, 11: estrone, 12:4-androstenediol, 13: testosterone, 14: estradiol, 15: dihydrotestosterone. veh, wells treated with vehicle only. *, steroids that increase an assay (p<0.05).

The two FPs in the FP_NLS_FP nuclear markers are separated only by an eighteen amino acid linker. Within any FP_NLS_FP, an FP that acts as a Förster Resonance Energy Transfer (FRET) acceptor could absorb emissions from the other FP (the donor) and thereby skew the em1/em2 ratio towards that of the ‘acceptor’ FP. The measured em1/em2 ratios (Fig. 4B, y-axis) agreed well with those predicted assuming no FRET (x-axis), although in many cases the properties of the FPs are so similar that, even if energy transfer would have been complete, it would have changed the em1/em2 ratios only slightly from that predicted assuming no FRET. Three FP_NLS_FP markers with distinct em1/em2 ratios (Fig. 4B, black arrows) were used in subsequent studies (Figs. 5, 6, 7, discussed below) to demonstrate that co-cultured cells labeled with those markers could be accurately distinguished by their characteristic em1/em2 ratios. We anticipate that this bar-code could be further expanded by identifying red FPs that occupy gaps remaining in em1/em2 ratios, such as between 0.8 and 1.1 (Fig. 4B, open arrow). The ability to predict reasonably well the em1/em2 ratios for various FP_NLS_FP nuclear markers (Fig. 4B) is expected to help identify FP_NLS_FP markers in that gap.

### Distinguishing Bar-Coded Cell Lines

For our remaining studies, we examined whether the bar-code would be useful for its intended purpose of distinguishing mixed, co-cultured cell lines. In theory, cells tagged with distinct FP_NLS_FP markers can, when mixed together (Fig. 5A), be distinguished by their distinct relative emissions in the em1 and em2 channels. Application of the bar-code depends on a very clean discrimination between the em1/em2 ratios for distinctly marked cells within a well. This relies upon the accurate measurement of em1 and em2 emission levels for every cell within a well. Accurate em1/em2 ratios also require accurate segmentation that minimizes the grouping of adjacent nuclei into a single object which would blend the em1/em2 ratios of different adjacent cells (Fig. 5A, black nuclei). We note that, with the demonstration of the effective application of the bar-code described below, we anticipate that the bar-code procedure even may be applied to help test how well new image segmentation algorithms improve the discrimination of adjacent objects.

To demonstrate the ability of bar-coding constructs to segregate co-cultured cell lines, we investigated two differentially-marked LNCaPC4-2 subclones that showed unique growth properties when grown separately. Our goal was to define whether that unique property would still be apparent if the cells were co-cultured in the same well and distinguished by the bar-code. Actinomycin D was previously identified in a screen as a drug blocking AR activity [31]. Longitudinal cell counting assays showed that the growth of two different LNCaP-C4-2 subclones was inhibited (p<0.001 for both lines) by incubation with 5×10^−8^ M actinomycin D (Fig. 5B). One LNCaP-C4-2 subclone had only ∼20% the cell numbers on Day 4 compared to Day 0 (Fig. 5B, mPlum_NLS_mPlum-marked subclone); This reduction in cell number was partially reversed by co-incubation with an AR activator (10^−9^ M DHT). Fortuitously, the second LNCaP-C4-2 cell subclone, marked with mCherry_NLS_mCherry, was less sensitive to actinomycin D (Fig. 5B, Day4/Day0 = 0.8). Although these differences caution about the potential selection biases in the generation of cell subclones, these unique properties were useful for confirming that the bar-code could be implemented.

To test the reliability of the bar-code for tracking the distinct growth properties of the two subclones, we first had to characterize how well em1/em2 ratios could properly score and distinguish mPlum_NLS_mPlum- and mCherry_NLS_mCherry-marked cells. Each subclone was grown in separate wells and imaged in both the em1 and em2 channels, at the same magnification (4x) used for the cell counting assays. For the mPlum_NLS_mPlum-marked subclone, a total of 88,766 objects were segmented; those objects had an em1/em2 channel ratio that averaged 1.099+/−0.059 (mean +/− sd). 168,157 of the mCherry_NLS_mCherry-marked objects were segmented with an average em1/em2 channel ratio of 0.579+/−0.031. We then set five standard deviations from each mean em1/em2 ratio as the margins for establishing whether an object would be scored as expressing mPlum_NLS_mPlum or mCherry_NLS_mCherry.

Of the 168,157 segmented objects collected from wells plated only with the mCherry_NLS_mCherry-marked cell line, 167,985 (99.90%) were within the em1/em2 ratios defined for mCherry_NLS_mCherry, 1 (0.00%) had em1/em2 ratios characteristic of mPlum_NLS_mPlum and 171 (0.10%) had other em1/em2 ratios. Of the 88,766 objects in wells plated only with the mPlum_NLS_mPlum-marked cells, 88,538 (99.74%) had em1/em2 ratios characteristic of mPlum_NLS_mPlum, 82 (0.09%) had em1/em2 ratios characteristic of mCherry_NLS_mCherry and 146 (0.16%) had other em1/em2 ratios. Therefore, the ability to accurately assign objects based on the bar-code was excellent even when, in subsequent studies described below, the em1/em2 scoring criteria was restricted to +/−3 sd.

### Unique Growth Characteristics Distinguished in Co-Cultured, Bar-Coded Cell Lines

With the excellent bar-code discrimination of the mPlum_NLS_mPlum- and mCherry_NLS_mCherry-marked cell lines, we examined the reliability of the bar-code to distinguish their unique actinomycin D responses when co-cultured. The two subclones were mixed, plated together, treated with DHT and/or actinomycin D and imaged by high throughput microscopy. Only 0.19% of the 282,029 objects in the 192 mixed wells were classified as debris (i.e., were not within the em1/em2 ratios used to define mPlum_NLS_mPlum or mCherry_NLS_mCherry cells). Figure 5C shows the distribution of em1/em2 ratios measured for all objects within one representative well in relationship to the fluorescence em1 channel fluorescence intensity measured for each object. The objects fall into two distribution patterns which show em1/em2 ratios within the boundaries established from the control wells (see previous section) that define the mCherry_NLS_mCherry- or the mPlum_NLS_mPlum-expressing cells (Fig. 5C, colored bars on x-axis). The scatter in em1/em2 ratio is greater within the mPlum_NLS_mPlum-expressing cells mostly because their measurements in the em2 channel are very low above background, which introduces inaccuracies (see Materials and Methods). Cells with higher signals above background in their em1 and em2 channels generally show tighter em1/em2 ratios (unpublished data).

When mixed together and separated on the basis of the bar-code, 5×10^−8^ M actinomycin D still selectively killed the co-cultured mPlum_NLS_mPlum-marked subclone (Fig. 5D) with only a modest effect on the mCherry_NLS_mCherry-marked cell line. Overall, the growth measurements for the co-cultured, bar-coded cells were similar to the same cells grown in separate wells for the four treatment conditions (p = 0.68), although there were some minor fluctuations most likely arising as statistical anomalies. The retention of the differential actinomycin D response for the two cell lines thus verified the fidelity of bar-code discrimination in co-cultured cells.

The well-to-well reproducibility required for high throughput screening usually is characterized by the Z’-factor score [32]. A difference between drug-treated and vehicle-treated wells that is more than twice that of the sum of three times the standard deviations for both measurements (Z’-factor>0.5) is generally considered sufficient for screening [32]. For wells co-plated with the two subclones then separated by the bar-code, the selective reduction in growth of the mPlum_NLS_mPlum-marked subclone upon treatment with Actinomycin D was reliably measured (Z’-factor = 0.64). That excellent reproducibility was similar (Z’-factor = 0.69) to that obtained when the actinomycin D-sensitive line was plated separately without need for separation by bar-code analysis. Thus, not only were the growth measurements the same for the co-cultured and individually cultures cells, the co-plated cells were separated by the bar-code so well that the stringent reliability of measurement required for high throughput studies was maintained.

These studies demonstrate that some drug screening campaigns can be shortened by using mixed cell lines. They also suggest ways to study the mutual effects on cell lines on each other, such as those mediated by paracrine signaling or well-established interactions amongst tumor, stroma and immune cells [33]–[35]. Thus, the co-culturing capability enabled by the bar-code also is likely to be useful for conducting studies and implementing screens in which cell-cell interactions may be the predominant biologic interest.

### Concurrent, Independent Assays Separated by the FP_NLS_FP Bar-Code

The cell counting studies showed that the bar-code effectively distinguished two different, co-plated cell lines. We next examined the utility of bar-coded cells for distinguishing different reporters co-cultured in a single well. Initially, we conducted these studies with two distinctly FP_NLS_FP-marked HeLa cell lines that expressed distinctly-regulated YFP-based reporters.

The AR is well-described to translocate into the cell nucleus upon the addition of androgen to the cell culture media [36]–[39], which has become the basis for many screens for both agonists and antagonists of AR action [31], [40]–[43]. A mCherry_NLS_mCherry-marked cell line was created that co-expressed a YFP-tagged wild-type AR. A mPlum_NLS_mPlum-marked cell line co-expressed a YFP-tagged mutant AR in which the threonine at amino acid 877 was changed to a serine. This T877S mutant AR, isolated from a prostate cancer tumor that continued to grow even when the patient’s testosterone levels were pharmacologically lowered, can be activated by certain steroids that only marginally activate the wild-type AR [44]–[46]. This differential response of the wild-type (AR_wt_-YFP) and mutant (AR_T877S_-YFP) ARs to some steroids was used to examine the effectiveness of the bar-code in distinguishing co-cultured assays.

The mCherry_NLS_mCherry-marked AR_wt_-YFP cell line and the mPlum_NLS_mPlum-marked AR_T877S_-YFP cell line were mixed and captured in the em1 and em2 bar-code channels along with a YFP ‘reporter’ channel. Figure 6A shows that the nuclei of the two cell lines are readily distinguished by their unique relative emissions in the em1 and em2 channels; the light blue-pseudo-colored nuclei represent the 1.06 ratio characteristic of mPlum_NLS_mPlum whereas the purple pseudo-colored nuclei represent the 0.55 ratio characteristic of mCherry_NLS_mCherry. The corresponding YFP ‘reporter’ image is shown in figure 6B. The cells within this representative image had been treated with 10^−7^ M estradiol, which is sufficient to activate translocation of AR_T877S_-YFP into the mPlum_NLS_mPlum-marked cell nuclei but insufficient to translocate AR_wt_-YFP in the mCherry_NLS_mCherry-marked nuclei. The estradiol-treated cells in which AR is predominantly nuclear (Fig. 6B, expanded inset) are those marked by mPlum_NLS_mPlum (Fig. 6A, light blue-pseudo-colored nuclei) and thus, those which express AR_T877S_-YFP. This demonstrated that the differential response of the two reporter cell lines was accurately discriminated using of the FP_NLS_FP bar-code.

To confirm those findings quantitatively, nuclear AR levels were averaged from 48 wells each (two fields per well) for each of the two reporter cell lines plated alone in separate wells or mixed together in a well and sorted on the basis of the bar-code. mCherry_NLS_mCherry-marked cells were identified as those with em1/em2 ratios between 0.4836 and 0.6252 (3 sds from the mean of 0.5544+/−0.0236 determined in wells expressing only mCherry_NLS_mCherry-marked cells). mPlum_NLS_mPlum-marked cells were identified as those with em1/em2 ratios between 0.9007 and 1.2244 (3 sds from the mean of 1.0626+/−0.0539 determined in wells expressing only mPlum_NLS_mPlum-marked cells). The analysis of the separately plated cells showed that only 8 of the 102,755 mCherry_NLS_mCherry cells would have been incorrectly assigned as mPlum_NLS_mPlum cells whereas 0 of the 70,252 mPlum_NLS_mPlum would have been incorrectly assigned as mCherry_NLS_mCherry cells.

When grown in the absence of any ligand (drug vehicle only), both cell lines showed low levels of nuclear YFP fluorescence for the AR_wt_-YFP and AR_T877S_-YFP reporters (Fig. 6C, veh.). Nuclear AR levels increased strongly in either cell line upon the addition of the androgens testosterone (Fig. 6C, Test.) or dihydrotestosterone (DHT). As expected, AR_T877S_-YFP responded robustly to progesterone (Prog.) and estradiol (Est.) whereas AR_wt_-YFP did not. Most importantly, those differential responses were similar if the cells were plated independently or mixed together in a single well then sorted by the bar-code. We did observe that the mCherry_NLS_mCherry-tagged cell line showed slightly, but consistently, higher measurements across all four treatment conditions (p<0.01) when collected from the co-cultured cells than when collected from the individually cultured cells. Because the mPlum_NLS_mPlum-tagged cells have higher levels of reporter expression for all treatment conditions, this may indicate some mis-assignment of mPlum_NLS_mPlum-tagged cells as mCherry_NLS_mCherry-tagged cells in the co-culture. Later examples (next section) however tend to indicate that the separation can be clean.

The Z’-factor scores that compare the well-to-well reproducibility of the vehicle-treated to the DHT-treated responses for AR_T877S_-YFP and AR_wt_-YFP, mixed and separated by the bar-code, were outstanding (0.840 and 0.835, respectively) and no poorer than for the cells plated separately in the same study (0.885 and 0.503). This demonstrated that it is possible to reliably mix together two different reporters in a drug or siRNA screen and then separate them to examine whether their responses are similar or distinct. As this could have been achieved also by simply tagging the mutant AR with YFP and the wild-type AR with, for example, CFP, the major utility of the nuclear bar-code resides in its potential to be used for multiple different co-plated assays (next section). However, the bar-codes described here also provide an advantage for separating two live cell assays, since it is preferable to avoid the damaging, near-uv light needed for the excitation of CFP or other blue-shifted FPs.

### Distinguishing Three Assays by the FP_NLS_FP Bar-Code

The studies of figures 5 and 6 showed that two bar-coded cell lines marked with mCherry_NLS_mCherry or mPlum_NLS_mPlum were accurately distinguished when mixed together. These two FP_NLS_FP markers are at opposite extremes of the em1/em2 ratios for the sixteen FP combinations we characterized (Table 4). To establish how well multiple bar-coded cells could be distinguished, we created three LNCaP-C4-2 human prostate cancer cell lines marked with distinct FP_NLS_FP markers. Each cell line co-expressed distinct YFP-based reporters. The mCherry_NLS_mCherry-marked subclone co-expressed a YFP-tagged wild-type AR (AR_wt_-YFP). The mRaspberry_NLS_mKate2-marked subclone co-expressed a YFP_NLS_YFP transcriptional reporter under the control of the AR-regulated mouse mammary tumor virus promoter (MMTV-YFP). The mPlum_NLS_mPlum-marked cell line co-expressed a YFP-tagged mutant AR (AR_T877A_-YFP).

The assay outputs for each cell line first were characterized independently to establish baseline measurements against which to evaluate the success of co-plating. Each of the three bar-coded cell lines was plated in 80 different wells and challenged with vehicle or 15 different natural steroids or their synthetic intermediates and metabolites (10^−8^ M each). Each treatment was conducted on five wells and two fields were collected per well. The intensity of background-subtracted YFP fluorescence in each cell nucleus was averaged for each field. Figures 7A–C show the mean +/− sd nuclear YFP values for all ten fields for each treatment. Measurements are shown for each of the three assays cultured separately (gray bars) or when all three assays were mixed together and separated by the bar-code (black bars) as detailed below.

The em1/em2 measurements for the three independently cultured mCherry_NLS_mCherry-, mRaspberry_NLS_mKate2- and mPlum_NLS_mPlum-marked cell lines were determined from a total of 81,027 cells as 0.560+/−0.025, 0.737+/−0.031 and 1.067+/−0.045, respectively. The em1/em2 margins used to identify the three different FP_NLS_FP-expressing cell types were defined as three standard deviations away from the mean obtained when those cell lines when plated by themselves. Analyses of the em1/em2 ratios from the separately plated cell lines (Table 5) showed that greater than 99.5% of all objects for each of the three cell lines would fall into the em1/em2 ratios characteristic of each cell-specific nuclear marker. Thus, the fidelity by which the bar-code would separate the co-cultured cell lines is expected to be very high. This high accuracy was confirmed when analyzing the bar-code-separated YFP measurements from the co-cultured assays. The responses obtained for the sixteen different treatments of each of the three different cell lines were the same (p = 0.24) for the co-cultured/bar-code separated wells (Fig. 7, black bars) and the wells in which the assays were plated independently (gray bars). Note that the LNCaP-C4-2 cell line expressing the AR_wt_-YFP assay had very poor Z’-factor scores insufficient for high throughput analysis. Still, this cell line was useful in the current demonstration that three cell types could be effectively discriminated by the bar-code. The distinct YFP responses verified the accuracy of the bar-code method for distinguishing different cell-based assays following their co-culture.

**Table 5:** Effective discrimination of three bar-coded cell-based assays.

FP_NLS_FP-markedLNCaP-C4-2 cell lines	# objectstotal	# objects inem1/em2 =	# objects inem1/em2 =	# objects inem1/em2 =	# otherobjects
		0.4841–0.6354*	0.6436–0.8299*	0.9331–1.2003*	
mCherry_NLS_mCherry	22,551	22,466	62	0	23
	(100%)	(99.62%)	(0.27%)	(0.00%)	(0.10%)
mRaspberry_NLS_mKate2	19,131	7	19,108	0	16
	(100%)	(0.04%)	(99.88%)	(0.00%)	(0.08%)
mPlum_NLS_mPlum	39,345	0	7	39,318	20
	(100%)	(0.00%)	(0.02%)	(99.93%)	(0.05%)

* Range of em1/em2 ratios within which the segmented nuclei were assigned, defined by mean +/−3 sd in em1/em2 ratios characteristic of each cell line.

**Figure 8:**
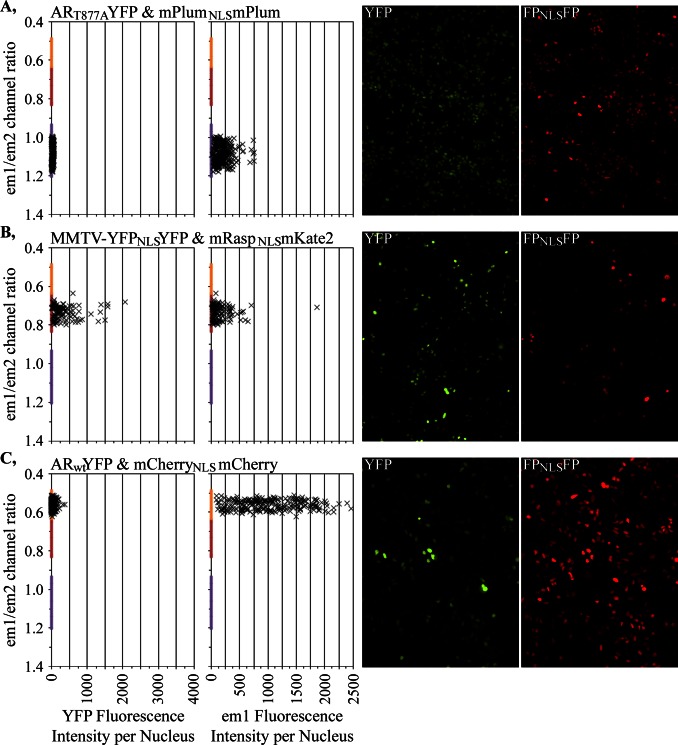
Distinct em1/em2 ratios characteristic for each of the three bar-coded LNCaP-C4-2 cell lines. **A–C,** Data from a representative field for each cell line. Each ‘x’ represents a YFP (left panel) or em1 (next panel) fluorescent intensity measurement from a single cell in relationship to the em1/em2 ratio measured for that cell. The images from which the measurements were obtained are shown. The representative fields for A and C were from cells treated with an androgen while the fields shown for B were vehicle treated because the MMTV-YFP assay intensity is much higher upon androgen treatment. The em1/em2 ratios used to assign a specific cell to a specific bar-code are shown as colored bars on the y-axis. The margins for those characteristic em1/em2 ratios were established as 3 sd away for all cellular measurements for the independently grown assays.

The em1/em2 measurements for individual cells within a representative field are shown in figure 8 for the three different cell lines studied in figure 7. Even when there was substantial heterogeneity in the amount of FP_NLS_FP expressed in each cell (Fig. 8C, em1 fluorescence), the em1/em2 measurements (with proper background subtraction) were consistent for cells across that wide range of expression levels. The consistency in em1/em2 measurement permitted the co-culture and successful bar-code separation of different cell lines with considerably different FP_NLS_FP expression levels (Figs. 7, 8). Exposure times must be set such that the lowest-expressing cells have sufficient intensity to be accurately measured in both em1 and em2, without saturating the intensities collected from the highest-expressing cells. It still is preferable to co-culture FP_NLS_FP-marked cell lines of similar, FP_NLS_FP intensities in at least the channel used for segmentation (em1 in our studies) since a segmentation setting applied across an image can provide slightly different margins for bright and dim objects. We prefer also cell lines expressing higher FP_NLS_FP levels because the higher signal to noise minimizes the improper segmentation of weakly fluorescent debris as nuclei.

### Relationships among Assay Outputs

The bar-code thus enables multiple assays to be assessed simultaneously upon exposure to precisely the same amount of drug. By contrast, if analyzing the same assays independently in separate studies, each assay can readily be exposed, through pipetting errors, to slightly different amounts of drug which would affect how well relationships amongst assays are classified. That consideration may be most helpful for screens in which replicate analyses are impeded by the amounts of time and cost needed to conduct different assays independently or when crucial materials are of limited availability.

For the current studies, the treatment response pattern for AR_T877A_-YFP level in the cell nucleus (Fig. 7A) paralleled that of the MMTV-YFP reporter (Fig. 7B). The strongest responses of both assays were to known androgens (compounds 12, 13 and 15) although many other steroids and steroid intermediates also activated both assays to lower levels. By contrast, AR_wt_ responded more selectively to the androgens or its immediate precursors (Fig. 7C).

The response of the T877A mutant AR to a broader array of steroids has long been known [45]–[47]. The endogenous AR in the LNCaP-C4-2 cells contains the T877A mutant which is why the MMTV promoter activity (Fig. 7B) paralleled nuclear translocation of the mutant AR_T877A_ (Fig. 7A) rather than the AR_wt_ (Fig. 7C). Note that some glucocorticoids, progestins and mineralocorticoids can activate MMTV promoter through endogenous receptors specific for those other steroids. Therefore, the nuclear translocation assay is very specific for the AR whereas the transcriptional reporter assay could be influenced by non-AR-relevant activities. The ability to distinguish such subtle cross-assay connections shows the utility of assessing multiple different assays together within a screening campaign. Through application of the bar-code, those connections can be faithfully retained and assessed in co-plated cells.

## Discussion

The FP_NLS_FP fusion protein adds to the current list of available nuclear markers, each with its relative strengths and weaknesses. Some differences in marking of mitotic and dying nuclei by FP_NLS_FP and DNA binding dyes were noted. Whether one opts for the FP_NLS_FP protein, FP fusions with other proteins or chemical staining as a nuclear marker will depend partly on the needs of each study. The FP_NLS_FP live cell marker also was useful for improving measurements of cell growth and death as a change in cell number within each well over time (Fig. 3, Table 2). Such longitudinal counting is not a specific property of the FP_NLS_FP marker and can be conducted with any FP-tagged nuclear marker or even lightly Hoechst-stained cells, provided that the markers themselves are not toxic to the cells [20], [27]–[28].

The FP_NLS_FP marker was not toxic at least to human prostate LNCaP-C4-2 cells. Those cells do not grow robustly and therefore might be considered a good test for FP_NLS_FP toxicity. But it remains unknown if the FP_NLS_FP would be non-toxic to every cultured cell type. As it is a marker that appears to be encapsulated by a nucleus rather than bound to a specific nuclear structure, the FP_NLS_FP marker theoretically might be less disruptive to the cell than some other currently available DNA-targeted markers.

We also demonstrate here the ability to distinguish slightly different variants of the FP_NLS_FP by virtue of their distinct properties in two emission channels Three studies were conducted on mixed cell populations tagged with the novel FP_NLS_FP bar-coded nuclear markers. Each of those studies demonstrated that co-cultured cells can be accurately identified by the unique fluorescence properties of the distinct FP_NLS_FP (Figs. 5, 6, 7). The ability to faithfully quantify multiple assays in one screen will help to broaden the types of measurements available and thereby improve the potential clinical and biological relevance of a primary screen [1]–[3].

In all our studies, the unique assay responses to a drug were faithfully maintained after co-culture. Indeed, that consistency in measurement provided strong evidence of the accuracy of the bar-code. We anticipate also that a major utility of the bar-code will be realized for screens of biologic responses that are modified upon the co-culture of different types of cells. It has become evident that cell-cell interactions, such as those of a tumor with its surrounding stroma or with infiltrating immune cells or of stem cells with surrounding tissues, is an important component of tumor or stem cell response that an effective drug must target [34]–[35], [48]–[51]. The FP_NLS_FP bar-code may prove to be essential for tracking different cell types co-cultured when attempting to reconstitute ‘pseudo-tissues’ for drug screening or even for low throughput studies of how cell mixtures affect biologic response.

For the most part, limitations in bar-code application are similar to those encountered when conducting a single assay. For example, if using the bar-code for high throughput screening, all cell lines mixed would have to show the very high well-to-well reproducibility required for successful screening. Thus, the LNCaP-C4-2 AR_wt_-YFP assay cell line examined (Fig. 7C) would be insufficient for use in a primary screen as the cell-to-cell variability is too poor for high throughput studies (Z’-factor = −1.4). By contrast, the mCherry_NLS_mCherry-marked HeLa AR_wt_-YFP assay (Fig. 6, Z’-factor ∼0.7), the mPlum_NLS_mPlum-marked LNCaP-C4-2 AR_T877A_-YFP assay (Fig. 7, Z’-factor ∼0.6) and the mRaspberry_NLS_mKate2-marked LNCaP-C4-2 MMTV-YFP assay (Fig. 7, Z’-factor ∼0.7) all have excellent well-to-well reproducibility and can be readily combined in a single bar-coded assay. Those assays also can be combined with day4/day0 cell counts (Fig. 5, Z’-factor ∼0.6) to establish the effects of any drug against multiple assay measurements in relationship to assay- or cell-specific effects of drugs on cell growth or toxicity. We already have successfully used that particular assay series to characterize hits from primary screens (unpublished data).

The ability to repetitively measure the FP_NLS_FP, live-cell nuclear marker over time also has other benefits over end-point assays that rely on cell fixation and staining. For example, when incubating cells for prolonged times with compounds and examining their effects on cell viability using an end-point assay, the cellular phenotypes associated with toxicity at earlier time points are not surveyed. The live cell bar-code assays permit cell counting/viability to be combined with one or more assays engineered as read-outs of mechanisms that may be sampled at multiple stages of the toxic response before the cells die. This additional information can provide an initial indicator of whether different hits identified in a screen are mechanistically similar or not.

Here we showed the clean separation of three different FP_NLS_FP markers based upon their characteristic em1/em2 ratios. There is room to expand beyond the three markers by developing other markers with em1/em2 ratios distinct from those used here (for example, mOrange_NLS_mOrange; unpublished data). Today, the application of the bar-code analysis for primary screens is limited mostly by the lack of integration of the bar-coding analysis package into the analysis/database software currently available on commercial high throughput microscopes. The application of the bar-code also could be improved by hardware upgrades in which a beam-splitter would allow the simultaneous collection of two or more emission channels at two or more cameras.

Eventually, we envisage full deployment of the bar-code technology also could be complemented by replacing the YFP reporter with various ‘green/yellow’ FP_NLS_FP reporters with unique ‘em3/em4’ ratios that would be matched with multiple ‘red’ bar-coded FP_NLS_FP markers. Our studies suggest that we may be able to collect up to four different red bar-coded FP_NLS_FP markers which, if combined with four different green bar-coded FP_NLS_FP reporters, could provide up to 16 different assays (plus cell growth/toxicity analyses for each) measured within a single well. Still, even the ability to distinguish three different bar-coded assays, demonstrated here, can broaden the parameters measured in a screen. That alone would substantially improve the identification and characterization of hits to improve the likelihood that a screen would define biologically or clinically relevant lead drugs or activities.

## Materials and Methods

### DNA Constructs

The cDNAs for the mCherry [52], mRaspberry [53] and mPlum [53] FPs were obtained with from the laboratory of Dr. Roger Tsien (University of California San Diego). The mKate2 [54] cDNA was purchased from Evrogen (Moscow, Russia). The FP_NLS_FP nuclear marker constructs were created by fusing pairs of PCR-amplified FP cDNAs in the combinations described in Table 4. The FP cDNAs were amplified using PCR primers that inserted the amino acids, including the SV40 nuclear localization sequence, indicated in figure 1A. The corresponding nucleotide sequences between the penultimate codon of the amino terminal FP and the methionine of the carboxy terminal FP are:

CCTCCAAAAAAGAAGAGAAAGGTAGAAGACCCCGGGGATCCACCGGTCGCCACC.

The fusion constructs were inserted into the expression vector backbone of the pEGFP-style constructs originally marketed by Clontech (Mountain View, CA, USA).

The MMTV-YFP reporter was made by first constructing a YFP_NLS_YFP expression vector as described above. The CMV promoter in that vector was excised by restriction with AseI and NheI and replaced with a 427 bp long fragment of the MMTV promoter PCR-amplified to contain AseI and NheI sites for that subcloning. The MMTV promoter sequence starts at 5′-AGTGGCT and ends at TGCGGCA-3′. Subsequent characterization showed that the YFP_NLS_YFP reporter used to construct this cell line had a deletion in the second of the tandem YFPs. The expression vectors for the YFP-labeled AR (wild-type, T877A and T877A mutants) were described previously as CFP-AR-YFP [46].

### Stable Cell Lines

Stable cell lines were subcloned from LNCaP-C4-2 cells purchased from ViroMed (Minnetonka, MN, USA) or from HeLa cells present within our laboratory. All stable cell lines were created by transfection of the DNAs into the cells by lipofectamine (Invitrogen, Carlsbad, CA, USA), followed by treatment with the selection agents listed below. Single colonies were evaluated by fluorescence microscopy for the appropriate intracellular distributions and uniformity of expression level of the FP-tagged reporters and nuclear markers. Cell lines expressing the reporters were further evaluated for appropriate androgen response when grown in the presence or absence of androgens. The selected stable cell lines were expanded and frozen. Cell lines were maintained in culture for less than 15 passages before new vials were thawed and propagated. The concentrations of selection drug used for maintenance were half those used for the initial selection (see below).

To generate cell lines expressing the CFP-AR-YFP and MMTV-YFP reporter, linearized vectors were used to help target integration to specific vector sites that did not disrupt expression of the reporters. Vectors were linearized by AseI restriction which cuts a single site immediately upstream of the CMV or MMTV promoters driving the expression of those reporters. A G418-resistance expression cassette in the CFP-AR-YFP and MMTV-YFP vectors was used to select for LNCaP-C4-2 or HeLa cell lines with an integrated expression cassette. G418 concentrations of 1600 µg/ml were used for selection.

FP_NLS_FP nuclear markers were introduced into the reporter-expressing cell lines. The FP_NLS_FP codons and associated CMV promoter and polyA signals were excised from the expression vectors by restriction with AseI and AflII. The isolated FP_NLS_FP expression cassette was co-transfected into the reporter expressing cells with an AseI-linearized pcDNA6/V5-His A vector that expressed the blasticidin-resistance marker (Invitrogen, Carlsbad, CA, USA). Cells resistant to 10 µg/ml blasticidin were selected and the expression of the intact FP_NLS_FP in cell nuclei was confirmed by fluorescence microscopy.

As a technical note, we found it necessary to excise the FP_NLS_FP expression cassette away from the sequences in those FP_NLS_FP vectors that express the G418-resistance gene. Otherwise cell lines were created in which both the reporter and the FP_NLS_FP expression cassettes, integrated at different chromosomal locations, were available to independently confer G418-resistance. In our experience the presence of two G418-resistance cassettes enabled the deletion of either the reporter or marker and led to instability in the cell lines.

### Cell Growth and Plating

HeLa and CHO cells were maintained in DME-H21 cell culture media supplemented with 5% calf serum and 2 mM glutamine. LNCaP-C4-2 cells were maintained in DME-H21 cell culture media supplemented with 10% fetal calf serum, 2 mM glutamine and 2 nM dihydrotestosterone. The androgen improved growth of LNCaP-C4-2 cells but had to be removed prior to experimentation. 24 hours before the initiation of cell plating for experimental studies, cells were washed extensively with ‘androgen-free’ media consisting of a 50:50 mixture of phenol-red-free DME-H21/Ham’s F-12 media supplemented with glutamine, and 5% fetal calf serum charcoal/dextran-stripped of steroids (HyClone SH30068.03, Thermo Scientific, Logan, UT, USA; in some experiments, we used newborn calf serum stripped three times within our laboratory). All subsequent experimental procedures were conducted in this androgen-free media.

Most studies were conducted with the HeLa or LNCaP-C4-2 cells stably expressing FP-tagged reporters and markers. LNCaP-C4-2 subclones ‘6+3’ and ‘44’ were used in the bar-code studies of figure 5. HeLa subclones ‘3-6-1’ and ‘T877S’ were used for the figure 6 bar-code studies. LNCaP-C4-2 subclones ‘F12’, ‘E01’ and ‘H’ were used for the bar-codes studies of figures 7 and 8. Studies comparing FP_NLS_FP distribution and growth measurements relative to Hoechst staining were conducted with the HeLa 3-6-1 subclone (Figs. 1, 2) and the LNCaP-C4-2 subclones F12, E01 and H (Fig. 3, Tables 1, 2). When using chemical dyes to count cells, wells were stained with 3 µg/ml of Hoechst 33342 in cell culture media for 40 minutes prior to imaging.

One day after changing to androgen-free growth media, the cells were collected by trypsinization, counted and, depending on the study and cell type, plated at 1000 to 2000 cells in 30 µl androgen-free media per well in a 384-well optical imagine plate (Greiner Bio-One 781091, Frickenhausen, Germany). Some wells were plated with media only (no cells) so that control images could be collected to ascertain and correct for, as described below, the contributions of media fluorescence and for the non-uniformity of image fluorescence across the field. Cells were treated the next day with 10 µl of androgen-free media containing 4x the final concentration of the indicated drugs; i.e., the studies were conducted at 1x final drug concentration in 40 µl final volume of androgen-free media.

For the studies in which the different FP_NLS_FP constructs were transiently expressed in CHO cells (Table 4), transfection was conducted with lipofectamine one day after changing to androgen-free media. The transiently transfected CHO cells were collected the following day and plated into 384-well Greiner Bio-One plates. Imaging of the CHO cells was conducted the day after plating.

### Imaging

For cell counting studies, Day 0 counts were conducted immediately after drug addition and again at later days under identical collection conditions. For examinations of AR activities, images were collected one day after drug addition. Image collection was conducted using an IXMicro High Throughput Microscope (Molecular Devices Corp., Sunnyvale, CA, USA). All filters and mirrors were obtained from Semrock, Inc. (Lake Forest, IL, USA). FP_NLS_FP images were collected with the FF01-575/15 excitation filter, the FF593-Di02 dichroic mirror and either the FF01-655-40 or FF01-628/40 emission filters, referred to as ‘em1’ or ‘em2’, respectively. Images for the YFP-based reporters were obtained using the 504/12 excitation filter, the FF440/520-Di01 dichroic mirror and the FF01-542/27 emission filter.

In our cell lines, western blots with an anti-AR antibody showed the CFP-AR-YFP (which is larger in size than the endogenous AR owing to the fused FPs) to be stably expressed at <5% the level of the endogenous AR in our LNCaP-C4-2 cell lines. This tracer level expression is ideal since the probe for AR activity is less likely to substantially affect the biology of the cells. However, expression level is so low that the poorly detectable CFP also is unable to be detected against the very high levels of background fluorescence in the CFP channel that originate from the culture media and serum. Thus, even though some cell lines expressed reporters in which AR was fused to both CFP and YFP, only YFP fluorescence was used to track the AR in those studies. We also avoided CFP collection since 1) energy transfer from CFP to YFP results in a loss of CFP signal that varies with different drugs and results in an under-representation of AR levels unless that energy transfer is determined and corrected for [55]–[56] and 2) excitation of CFP requires higher-energy light sources that is damaging to live cells [27]–[28] which could introduce errors into our proliferation studies where the same cells are re-imaged on subsequent days.

The image collection times varied with cell assay and objective used but generally were set so that em1 emissions of the most highly expressed FP_NLS_FP-expressing cells would average around 1000 units on a 12-bit scale with no pixels saturated in either the em1 or em2 channels. All images were collected with no pixel binning to permit optimal segmentation of the nuclei at image analysis. For cell counting studies, all image collection and image analysis parameters were identical on Day 0 and subsequent days.

### Image Analysis and Background Collection

The em1 image, which represents fluorescence from FP_NLS_FP expressed in the cell nuclei, was used to identify cell nuclei by automated image segmentation. Segmentation was conducted with the ‘Count Nuclei’ program of the IXMicro analysis software (Molecular Devices Corp). The amounts of fluorescence in each segmented nucleus in each channel (YFP, em1, em2) were saved to a database together with other quality control information such as cell area. For the current studies, we used Microsoft Excel (Redmond, WA, USA) to create macros that segment out different cell types (together with the amounts of YFP reporters in the nuclei of each cell) based upon their em1/em2 ratios. The em1/em2 ratios characteristic for each cell type were determined from the averages collected from tens of thousands of measurements from individual cell lines grown as monocultures (i.e. not mixed with other bar-coded cell lines). Identified cells with saturated pixels also were noted so that their em1/em2 characterization could be flagged as questionable and their results eliminated from further analysis.

To obtain accurate and reproducible em1/em2 ratios of the bar-code markers, it is crucial to ensure that fluorescence amounts not originating from the FP_NLS_FP are accurately removed prior to calculating the ratio. This background originates from a number of sources including camera noise and fluorescence from the media and/or the drugs added to the media. The walls of some plates also reflect background light back into the image; for accuracy, it is important to ensure that your image collections are not subject to such reflections. A more common source of non-uniformity in background fluorescence originates with the instrument’s optics. To correct these non-uniformities, we typically create ‘background images’ for each channel collected under the same image collection conditions from wells in which media only was plated (i.e., no cells). These background images also will contain fluorescence originating with camera noise and media fluorescence. The background images were typically averaged from 40 fields selected to show no evidence of any unusual fluorescence debris in each channel.

For every set of em1, em2 and YFP images from each field, the em1, em2 and YFP background images were first subtracted. For the most part, since the em1 and em2 ‘red’ fluorescent channels contained very little fluorescence from the media (which contained no phenol red), the subtraction of the background image alone provided a reasonably good background subtraction. However, we routinely saw some well-to-well variations in em1 and em2 background fluorescence, typically +/−1 to 3 units on the 12-bit (0–4095) intensity scale. Thus, procedures (described in the next paragraph) were used to define those small deviations from the background image so that they could be corrected for and improve em1/em2 ratio calculation particularly in nuclei having low fluorescence signal above the background noise. This correction becomes very important when running the analyses in screening mode as we have observed large numbers of compounds to have some level of fluorescence when added to the media. We also note that autofluorescence from the cells themselves would not be corrected by the methods below. Fortunately, that autofluorescence is negligible in the ‘red’ em1 and em2 channels used here and did not need to be corrected.

In order to define the additional background correction amounts, the areas where no cells were present first were identified by running a segmentation protocol to identify all objects, even those that are small and of very low fluorescence above local background. That protocol was different than the more stringent protocol used to restrict segmentation to larger, brighter nuclei. The objects identified were expanded by three pixels in all directs to create a ‘mask’ of all cells and debris. The fluorescence levels in the areas outside of that mask were determined for all of the em1, em2 and YFP channels. That constituted the ‘background’ measured in each field containing cells. That same mask was applied to define the em1, em2 and YFP backgrounds in the same area of the background image. The amount of fluorescence in the cell-containing image above/below that in the background image was subtracted/added to obtain the final fluorescence corrections. All em1, em2 and YFP measurements in the cell mixing studies used those corrections.

### Statistical Analysis

FP_NLS_FP-marked cell lines were cultured separately, or together with other cell lines and identified by the bar-code, under treatment conditions that generated unique responses (Figs. 5, 6, 7). Two-way analysis of variance were used to examine the crucial question of whether the measurements for all treatment groups were similar for individually-cultured and bar-code separated cells. The effectiveness of bar-code discrimination was evaluated by using different treatments to obtain different assays results. Treatments that were statistically significant relative to specific controls were established by unpaired t-tests and are indicated by symbols described in the figure legends.
